# A Used Ball of Cotton Wool as a Source of Nosocomially-Acquired Hepatitis C Infection

**Published:** 2010-03-01

**Authors:** Clement Ibi Mboto, Iquo A. Takon, John Ele Ndem

**Affiliations:** 1 Department of Microbiology, Faculty of Science, University of Calabar, Calabar, Nigeria; 2 Department of Pathology, General Hospital, Akamkpa-Cross River State, Nigeria

**Keywords:** Cotton Balls, HCV, Nosocomial

## Abstract

**Background and Aims:**

An error involving the reuse of the same ball of cotton wool in stopping blood flow after venous blood collection from five antenatal women prompted further investigation and follow-up studies to rule out nosocomially-acquired blood borne viruses.

**Methods:**

The five women were screened for antibodies to the human immunodeficiency virus (HIV), hepatitis C virus (HCV) and hepatitis B surface antigen (HBsAg), using enzyme-linked immunosorbent assay (ELISA) /kits Murex HIV–1- ,2,0 (Murex Biotech, UK); ORTHO HCV 3.0 ELISA Test kit (Ortho Clinical Diagnostics, USA); and QUADRATECH CHECK 4-HBs one-step generation test kit (VEDALAB, France) respectively. The tests were repeated in 2005 on the five women, their husbands and twenty children, aged nine months to seven years borne by all the women within the period. Anti-HCV was detected in one out of the five women at the initial stage of the error (1997). No anti-HIV or HBsAg was found in any of the women. A repeat screening for anti-HIV, anti-HCV and HBsAg carried out seven years later (2005) on the five women, their husbands and twenty children aged nine months to seven years borne by all the women within the seven years revealed an HCV sero-conversion in two additional women. No anti-HCV or anti-HIV nor HBsAg was detected in any of the women, their spouses or their 20 offspring.

**Results:**

Anti-HCV was detected in one out of the five women at the initial stage of the error (1997). No anti-HIV or HBsAg was detected in any of the women. A repeat re-evaluation revealed an HCV sero-conversion in two additional women. No anti-HCV or anti-HIV nor HBsAg was detected in any of the women, their spouses or any of their 20 screened offspring.

**Conclusions:**

This study provides evidence for the nosocomial transmission of HCV through the use of a contaminated ball of cotton wool. It also confirms the poor efficiency of sexual and vertical transmission of HCV and calls for improved hospital facilities and the use of skilled staff to perform essential duties.

## Introduction

Hepatitis C is strictly a human infectious disease of the liver that is often asymptomatic [1]. Once established, chronic infection proceeds and can progress to fibrosis and cirrhosis after a period of many years of apparency. Globally it is estimated that 200 million people are infected with the virus with a larger percentage in Sub-Saharan Africa [1].

Unlike the developed countries where hepatitis C virus has mainly been spread by blood-to-blood contact through transfusion of unscreened blood or blood products (before the emergence of HCVspecific diagnostic tests), and now predominantly via injected-drug use or sexual exposure [2]; the primary sources of HCV infection in developing countries are unsterilized injection equipment and the infusion of inadequately screened blood and blood products [1]. Some studies have shown that people can be exposed to HCV via inadequately or improperly sterilized medical or dental equipment through accidental exposure to blood by means of accidental needle sticks or blood spatter to the eyes or open wounds.

Given the high prevalence of HCV in Sub-Saharan Africa and the establishment of the fact of infrequent intravenous drug use in Sub-Saharan Africa and of the low efficiency of sexual transmission of HCV, some authors have suggested iatrogenic causes as a possible mode of HCV transmission in the region [2][3]. This prompted the investigation of an incident in which a trainee medical laboratory assistant in March, 1997 reused the same ball of cotton wool to stop bleeding in five antenatal women after venous blood collection.

## Materials and Methods

### Study site and subjects

The study site for this work is the General hospital,Akamkpa, in the Cross River State of Nigeria; the preliminary subjects were antenatal women seen in a routine medical checkup. The follow-up subjects were all located within the local government area of Akamkpa.

### Collection of blood samples

Routine venous collection of blood from antenatal women for blood group and genotype determination and detection of antibodies to HIV was interrupted after five women aged 19 to 42 years (mean 29.7 years) were consecutively found to have had their post-venous-blood-collection bleeding stopped using the same ball of cotton wool. The five women were counselled and informed of the error and their informed consent obtained for follow-up investigations.

### Follow-up investigations

As a follow-up to address the possibility of cross-transmission of blood-borne viruses [4] among the women, approximately 5ml of venous blood was collected from each woman and labelled RER, EAY, ABE, OKB, DKB( after their initials) Each blood sample was screened in turn for human immunodeficiency virus (HIV), hepatitis B surface antigen (HBsAg) and Hepatitis C (HCV).A screening test for Anti-HIV was carried using enzyme-linked immunosorbent assay (ELISA) kits Murex HIV–1,2,0 (Murex Biotech, UK) [5]; Hepatitis B surface antigen (HBsAg) was screened for, using QUADRATECH CHECK 4-HBs onestep generation test kit (VEDALAB, France) [6]; while anti-HCV was screened for, using the secondgeneration enzyme-linked immunoassay (Monolisa -R, Sansofi, Pasteur; France).

### Repeat tests

The exercise could not be repeated until September 2005. In this case venous blood was collected from all five women who were local farmers/housewives, their spouses and the twenty children, aged 1-7 years borne by the women between 1997 and 2005. The same type of test kits were employed for HBsAg and HIV as in 1997, except for HCV, for which the ORTHO HCV 3.0 ELISA Test kit (Ortho Clinical Diagnostics, USA) [7][8] was used in place of the second-generation enzyme-linked immunoassay (Monolisa -R, Sansofi, Pasteur; France).

## Results

During the first screening exercise in March,1997, anti-HCV was detected in one of the women (a 20% prevalence rate) who the laboratory assistant admitted to be the second bled in sequential order of the five women (Table 1). No HBsAg or anti-HIV was detected in the five women.

The re-evaluation exercise carried out in September 2005 revealed the presence of anti-HCV in three out of the five women (a 60% prevalence) and none in their spouses or their 20 children, including eleven children from the three HCV- positive women. Similarly, no hepatitis B surface antigen (HBsAg) or antibody HIV was detected in any of the women,their spouses or the screened children (Table 2). An interview with the women showed that none of them had been transfused within the past ten years.

**Table 1.** Order by which antenatal care (ANC) women were bled in relation to their HCV status.

**Sequential order of initial blood collection^a^**	**HCV status as to March, 1997 (n=5)**	**Anti-HCV test result September, 2005 (n=5)**
AGH	Negative	Negative
KLO	Positive	Positive
TIL	Negative	Positive
NAS	Negative	Positive
GTE	Negative	Negative

^a^ Patients initials

**Table 2:** Summary of test results of follow-up study of pregnant women, their spouses and children according to age and sex.

**Age**	**Sex**		**Results**		
**(Years)**	**Male (n=17; %)**	**Female (n=13 ;%)**	**HIV Positive**	**HBsAg Positive**	**HCV Positive (n=3 ;%)**
0-7	13(76.5)	7(53.8)	0	0	0
8-15	0	0	0	0	0
16-22	0	1(7.7)	0	0	0
23-30	1(5.9)	1(7.7)	0	0	0
31-37	0	1(7.7)	0	0	1(33.3)
≥38	3(17.6)	3(23.1)	0	0	2(66.7)

## Discussion

The seroprevalence of HCV in Nigeria is unclear, and its epidemiology, particularly in women and children, is yet to be established. Some recent studies have reported an HCV prevalence of 5% in apparently healthy blood donors [9] and 14.1% in diabetic patients10. However, unlike some developed countries where HCV transmission is predominantly associated with the sharing of contaminated needles and other drug paraphernalia by intravenous drug users [2], the precise modes of transmission of HCV in Nigeria remains unclear [1]. Some authors have also suggested iatrogenic causes as a possible mode of HCV transmission in the region [2][3]. Furthermore, there is a paucity of data on the contributory role of household transmission, needle stick injuries, contaminated medical equipment, and blood spills in health care settings in the distribution of the virus in the country. Available data however, shows that each year, the reuse of injection equipment may cause 2 million infections with hepatitis C virus (HCV) worldwide [2].

In many developing countries inadequacies in health care settings have often resulted in the recycling of some disposable items which may include needles and gloves. This enhanced by the dearth of skilled staff poses a major problem in the diagnosis and management of patients in many health care settings. In this study, the detection of antibodies to HCV in the two women who preceded the first woman screened as anti-HCV positive in 1997 provides evidence of a possible cross-infection emanating from the reused ball of cotton wool. Although the viral genotypes were not characterized (Njouom et al., 2003) [3] to determine their relatedness, the absence of a history of blood transfusion within the past ten years among the women rules out this mode as a possible means of spreading the infection. Similarly, the non-detection of antibodies to HCV in the spouses of the HCV seropositive women more than seven years after their acquisition of the infection also confirms the poor level of efficiency of sexual transmission of the virus [10][11]. Although the sexual transmission of HCV is well established, its efficiency of transmission has been shown to be poor [10]. Furthermore, the non-detection of anti-HCV among the eleven [11] surviving children borne by the three women with HCV antibodies also confirms the low level of vertical transmission of the virus [12][13].

Some studies have shown that HCV can survive outside the body for up to 3 months, and its RNA remains stable at room temperature for several hours [14]. In addition, HCV has been shown to withstand a high level of acidity for hours. These features are all likely to have favoured the acquisition of the virus by means of the contaminated cotton wool.

## Conclusions

This study implicates a contaminated ball of cotton wool as a source of transmission of HCV and supports reports of iatrogenic causes as the main source of transmission of blood borne viruses in most developing countries. It also provides evidence in support of the poor sexual [10][11] and vertical transmission [13] of HCV and makes need for the use of skilled staff and improved laboratory facilities in developing countries.

## References

[1] Mboto CI, Davies A, Fielder M, Jewell AP (2006). Human immunodeficiency virus and hepatitis C co-infection in sub- Saharan West Africa. Br J Biomed Sci.

[2] Madhava V, Burgess C, Drucker E (2002). Epidemiology of chronic hepatitis C virus infection in sub-Saharan Africa. Infect Dis.

[3] Njouom R, Pasquier C, Ayouba A, Gessain A, Froment A, Mfoupouendoun J, Pouillot R, Dubois M, Sandres-Sauné K, Thonnon J, Izopet J, Nerrienet E (2003). High rate of hepatitis C virus infection and predominance of genotype 4 among elderly inhabitants of a remote village of the rain forest of South Cameroon. J Med Virol.

[4] (1998). Recommendations for prevention and control of hepatitis C virus (HCV) infection and HCV-related chronic disease. Centers for Disease Control and Prevention. MMWR Recomm Rep.

[5] Beelaerta G, Vercauterenb G, Fransena K, Mangelschotsa M, De Rooya M, Garcia-Ribasa S, van der Groen G (2002). Comparative evaluation of eight commercial enzyme linked immunosorbent assays and 14 simple assays for detection of antibodies to HIV. J Virol Methods.

[6] Wolters G, Kuijpers LP, Kacaki J, Schuurs AH (1977). Enzymelinked immunosorbent assay for hepatitis B surface antigen.. J Infect Dis.

[7] (1991). Public Health Service inter-agency guidelines for screening donors of blood, plasma, organs, tissues, and semen for evidence of hepatitis B and hepatitis C. MMWR Recomm Rep.

[8] Conry-Cantilena C, VanRaden M, Gibble J, Melpolder J, Shakil O, Viladomiu L, Cheung L, DiBisceglie A, Hoofnagle J, W. Shih J, Kaslow R, Ness P, J. Alter H (1996). Routes of infection, viremia, and liver disease in blood donors found to have hepatitis C virus infection. N Engl J Med.

[9] Jeremiah ZA, Koate B, Buseri F, Emelike F (2008). Prevalence of antibodies to hepatitis C virus in apparently healthy Port Harcourt blood donors and association with blood groups and other risk indicators. Blood Transfus.

[10] Gordon SC, Patel AH, Kulesza GW, Barnes RE, Silverman AL (1992). Lack of evidence for the heterosexual transmission of hepatitis C. Am J Gastroenterol.

[11] Hallam NF, Fletcher ML, Read SJ, Majid AM, Kurtz JB, Rizza CR (1993). Low risk of sexual transmission of hepatitis C virus. J Med Virol.

[12] Bortolotti F, Resti M, Giacchino R, Azzari C, Gussetti N, Crivellaro C, Barbera C, Mannelli F, Zancan L, Bertolini A (1997). Hepatitis C virus infection and related liver disease in children of mothers with antibodies to the virus. J Pediatr.

[13] Njouom R, Pasquier C, Ayouba A, Tejiokem MC, Vessiere A, Mfoupouendoun J, Tene G, Eteki N, Lobe MM, Izopet J, Nerrienet E (2005). Low risk of motherto- child transmission of hepatitis C virus in Yaounde, Cameroon: the ANRS 1262 study. Am J Trop Med Hyg.

[14] Thompson SC, Boughton CR, Dore GJ (2003). Blood-borne viruses and their survival in the environment: is public concern about community needlestick exposures justified?. Aust N Z J Public Health.

